# Rethinking Regenerative Medicine: A Macrophage-Centered Approach

**DOI:** 10.3389/fimmu.2014.00510

**Published:** 2014-11-04

**Authors:** Bryan N. Brown, Brian M. Sicari, Stephen F. Badylak

**Affiliations:** ^1^McGowan Institute for Regenerative Medicine, University of Pittsburgh, Pittsburgh, PA, USA; ^2^Department of Bioengineering, University of Pittsburgh, Pittsburgh, PA, USA; ^3^Department of Surgery, University of Pittsburgh, Pittsburgh, PA, USA

**Keywords:** regenerative medicine, biomaterials, host response, foreign-body reaction, stem cells, macrophages

## Abstract

Regenerative medicine, a multi-disciplinary approach that seeks to restore form and function to damaged or diseased tissues and organs, has evolved significantly during the past decade. By adapting and integrating fundamental knowledge from cell biology, polymer science, and engineering, coupled with an increasing understanding of the mechanisms which underlie the pathogenesis of specific diseases, regenerative medicine has the potential for innovative and transformative therapies for heretofore unmet medical needs. However, the translation of novel technologies from the benchtop to animal models and clinical settings is non-trivial and requires an understanding of the mechanisms by which the host will respond to these novel therapeutic approaches. The role of the innate immune system, especially the role of macrophages, in the host response to regenerative medicine based strategies has recently received considerable attention. Macrophage phenotype and function have been suggested as critical and determinant factors in downstream outcomes. The constructive and regulatory, and in fact essential, role of macrophages in positive outcomes represents a significant departure from the classical paradigms of host–biomaterial interactions, which typically consider activation of the host immune system as a detrimental event. It appears desirable that emerging regenerative medicine approaches should not only accommodate but also promote the involvement of the immune system to facilitate positive outcomes. Herein, we describe the current understanding of macrophage phenotype as it pertains to regenerative medicine and suggest that improvement of our understanding of context-dependent macrophage polarization will lead to concurrent improvement in outcomes.

## Introduction

The macrophage has long been known to play an important role in the tissue remodeling response which occurs following injury. In brief, macrophages arrive at the site of tissue injury 24–48 h post-injury, serve as phagocytes clearing the wound bed and initiating the processes that lead to the default outcome of scar tissue formation (1, 2). However, only recently it has been recognized that macrophages can have positive impacts upon tissue remodeling following injury (3–9). While the specific mechanisms by which macrophages direct tissue remodeling responses remain a subject of ongoing research, it has been suggested that a transition from a pro-inflammatory (M1) phenotype to a more regulatory or anti-inflammatory M2 phenotype is a key aspect of tissue remodeling which promotes functional outcomes as opposed to scar tissue formation.

A correlation of macrophage phenotype with functional recovery in wound healing has been suggested for more than two decades (9). With the introduction and general acceptance of the M1/M2 phenotypic dichotomy (10), correlation of macrophage polarization states and functional recovery has now been reported in several other tissues and organ systems and represents an area of increasing interest for those in the area of wound healing and regeneration. The central dogma of this macrophage-centered approach is that treatments which facilitate an efficient and timely switch from a pro-inflammatory to an anti-inflammatory and regulatory phenotype, will logically promote functional tissue remodeling over scar tissue formation.

The M1/M2 paradigm has been widely studied in the context of disease pathogenesis, particularly cancer, for more than two decades (11–13). The participation of M1 and M2 macrophages in a diverse set of diseases including atherosclerosis, endometriosis, and pulmonary fibrosis is also now recognized (2, 12, 14–18). In addition, there is evidence for the importance of macrophages in tissue and organ development and in processes such as limb regeneration in the axolotl (19, 20). Loss of macrophages during these processes leads to defects in development or retardation of the regenerative process in the axolotl. Similar findings have been reported in other regenerative species including zebrafish, where ablation of macrophages results in defects in fin regeneration following injury (21). This ability to promote a regenerative response is lost in higher order species with increasing complexity of the immune system, having been replaced with a default mechanism of “rapid resolution” (i.e., scarring). While the mechanisms which underlie the loss of regenerative potential remain largely unknown, a better understanding of the role of the innate immune system in the regenerative process of lower organisms may provide targets for regeneration strategies in humans (22).

Regenerative medicine approaches to tissue reconstruction or organ replacement seek to restore the form and function of lost, damaged, or diseased tissues. These approaches logically rely upon our understanding of wound healing, development, and regeneration as guideposts for design. These approaches may incorporate one or more biomaterials, biologically active molecules, and/or cell sources. Recent advances in these areas have enabled highly innovative and promising therapies, but translation of such strategies, without exception, requires in depth investigation and understanding of the host response following delivery.

The purpose of the present review is (1) to provide rationale for a macrophage centric approach to tissue reconstruction; and (2) to give an overview of the current state-of-the-understanding of the implications associated with host macrophage responses in regenerative medicine.

It should be noted that the description of macrophages as having either an M1 or M2 phenotype is a simplification of the *in vivo* reality. Though it is now clear that M1 and M2 macrophages each play distinct roles in tissue remodeling following injury, the inflammatory process which occurs following injury is dynamic both spatially and temporally and macrophages may express transitional phenotypes. Logically, these cells will also express functions such as phagocytosis, antigen presentation, and effector molecule production to differing degrees during the inflammation and remodeling process. For the purposes of simplicity and general discussion, and as the M1/M2 terminology are used ubiquitously throughout the literature, we describe macrophage phenotype as M1 and M2 in the below examples with further discussion of the spectrum of possible phenotypes and their potential roles in regenerative medicine thereafter.

## A Macrophage Centric Approach

There is evidence for both pathogenic and protective roles of macrophages in many biologic processes (12, 23). It is well understood that uncontrolled inflammation can be a detrimental process (e.g., inflammatory bowel disease, rheumatoid arthritis). However, an organized and well regulated macrophage response has been shown to be a determinant of tissue remodeling following injury, with the potential for positive outcomes and functional recovery. The key role of macrophages in functional recovery following injury suggests that methods which are capable of modulating the macrophage response in a controlled, reproducible, and well-defined manner, may also meet with improved outcomes in regenerative medicine applications. Below, we review the role of macrophages in the response to tissue injury and the subsequent remodeling process in three different tissue environments as a baseline from which to understand the potential role of macrophages in regenerative medicine approaches to tissue reconstruction and to provide the rationale for a macrophage centric approach.

### The M1/M2 paradigm during the skeletal muscle injury response

The role of the M1/M2 paradigm during the skeletal muscle injury response is relatively well characterized. Skeletal muscle tissue possesses inherent regenerative capacity following acute injury such as exercise-induced trauma. The capacity of muscle tissue to regenerate relies heavily upon a population of normally quiescent muscle specific progenitor cells, referred to as “satellite cells,” and their interactions with inflammatory cells that infiltrate the injured muscle microenvironment (24, 25). An efficient skeletal muscle injury response which successfully restores the injured muscle tissue requires satellite cell progression through a structured process of activation including proliferation and subsequent maturation into committed myoblasts, myoblast alignment, and finally, fusion and differentiation into new contractile skeletal muscle myotubes (26–29). This carefully regulated process of satellite cell differentiation is controlled, in large part, by the activity of an orchestrated heterogeneous inflammatory response consisting predominantly of M1 and M2 polarized macrophages (30, 31).

Following acute skeletal muscle injury, one of the earliest events is the infiltration of the damaged tissue by inflammatory cells. Neutrophils comprise the initial wave of cells to enter the damaged muscle tissue and reach elevated levels as soon as 2 h post-injury and maximum numbers after 6–24 h (30, 32, 33). Neutrophils phagocytose host necrotic cellular or bacterial debris and propagate a pro-inflammatory response through the release of reactive oxygen species and T-helper (Th)1 associated pro-inflammatory cytokines, which recruit monocytes and macrophages. However, neutrophil numbers decline through apoptosis and the neutrophil response is generally resolved by 3–4 days post-injury (30). Monocyte-derived macrophages recruited to the damaged tissue shortly after neutrophil infiltration represent the predominant immunologic participant in the skeletal muscle injury response thereafter.

Monocytes originate in the bone marrow and express chemokine receptors and adhesion molecules which facilitate their extravasation and migration from the blood to the injured skeletal muscle tissue site where they differentiate into macrophages (34, 35). Tissue resident macrophages normally found within the skeletal muscle microenvironment are also likely to play a role. However, the relative contributions of tissue resident macrophages as compared to circulating macrophages to the tissue remodeling process remain unknown. Immediately after injury, infiltrating macrophages become polarized toward a pro-inflammatory or M1 phenotype. The mechanisms behind this M1 activation remain only partially understood and include macrophage exposure to pro-inflammatory cytokines (i.e., IFN-γ, TNF-α) and/or necrotic cellular or bacterial debris (30, 35–37). M1 macrophages within the injured muscle microenvironment phagocytose necrotic muscle debris and participate in a transient pro-inflammatory response, reaching elevated levels at 24 h post-injury and maximum numbers after 2 days (30, 35). In addition to producing large amounts of pro-inflammatory cytokines (i.e., TNF-α, IL-1β, IL-12), M1 macrophages process and present antigen and express high levels of iNOS which facilitates NO production (38, 39).

After 2 days, macrophages participating in the remodeling of injured skeletal muscle show a transition from the pro-inflammatory M1 to the immunoregulatory and anti-inflammatory M2 phenotype. The mechanisms behind this M1 to M2 phenotypic switch remain only partially understood but include exposure of M1 macrophages to increased IL-10 concentrations from skeletal muscle at 48 h post-injury (40, 41); M1 macrophage mediated phagocytosis of apoptotic, as opposed to necrotic, cells (40, 42); and exposure of M1 macrophages to degradation products from extracellular matrix (ECM) (43). M2 macrophages reach peak numbers within areas of injured muscle at 4 days post-injury and remain a predominant cell-type present in the remodeling muscle microenvironment for several days (44, 45). M2 macrophages facilitate resolution of inflammation through the release of anti-inflammatory cytokines (i.e., IL-10, IL-13), which deactivate pro-inflammatory cell phenotypes and promote tissue remodeling and repair (35, 44, 45).

This transition of the initial response dominated by M1 macrophages to a more M2 dominated population following acute muscle injury facilitates skeletal muscle remodeling and is required for efficient and compete functional restoration. Specifically, the pro-inflammatory products of M1 macrophages promote the activation and expansion of quiescent muscle satellite cells within the tissue injury site (35, 38, 46, 47). For example, TNF-α produced in large quantities by M1 macrophages represents a well-accepted mitogen for satellite cell-derived skeletal muscle myoblasts (31, 48). Following satellite cell and myoblast expansion, paracrine signals from M2 macrophages facilitate the alignment, fusion, and differentiation, of these skeletal muscle progenitor cells. For example, IL-10, an immunomodulatory cytokine produced by M2 macrophages, is myogenic for skeletal muscle progenitor cells (4, 31).

The participation of a heterogeneous population macrophages following muscle injury is highly regulated. For example, the perturbation or prolongation of either the M1 or M2 macrophage population during the skeletal muscle injury response results in impaired skeletal muscle regeneration. Depletion of phagocytic leukocytes, including macrophages, prior to toxin induced skeletal muscle injury blocks the removal of cellular debris and impairs regeneration (31). Depletion of macrophages at the time of injury prevents the participation of M1 macrophages in the early response and therefore shows their importance to muscle regeneration (31, 49). Furthermore, immediately following injury, skeletal muscle shows decreased activity of muscle specific transcription factors in TNF-α knockout animals when compared to their wild-type counterparts (50, 51), suggesting that TNF-α from M1 macrophages promotes the early or proliferative stage of myogenesis. However, prolonging TNF-α activity beyond the early proliferative stage of myogenesis has deleterious effects. While TNF-α promotes skeletal muscle precursor cell mitogenesis, it also inhibits myogenesis (52–54).

The participation of M2 macrophages is also required for the skeletal muscle injury response. As stated above, these immunomodulatory cells drive the late or differentiation stage of myogenesis. For example, when macrophages were deleted after 2 days post-injury, a time point consistent with the transition to an M2 macrophage response, myoblast differentiation, and subsequent regeneration was impaired (55). Furthermore, transgenic animals unable to mount a M2 macrophage response show an accumulation of proliferative myoblasts and a lack of myogenic differentiation following injury (7). These studies show the importance of a present, yet regulated, M1 to M2 phenotypic transition of macrophages for efficient skeletal muscle regeneration.

### The M1/M2 paradigm during cutaneous wound healing

Adult mammalian cutaneous wound healing is another highly regulated process that follows a sequence of events comprising the following three interdependent and overlapping phases: (1) the inflammatory phase; (2) the granulation tissue formation and wound contraction phase; and (3) the matrix deposition and tissue remodeling phase (56, 57). Multiple studies have now demonstrated distinct macrophage phenotypes associated with each of these phases and with remodeling outcomes following injury.

The onset of wound healing, designated the inflammatory phase, can be further sub-divided into an early and late inflammatory phase. Immediately following injury, hemostasis provides a provisional matrix for cell migration. During the early inflammatory phase, which occurs at 1–4 days post-injury, neutrophils and monocyte-derived macrophages respond to pro-inflammatory signals released from the wound microenvironment including growth factors, cytokines, damage associated molecular patterns (DAMPS), and pathogen associated molecular patterns (PAMPS) (58). These pro-inflammatory effector molecules along with the presence of necrotic cellular and bacterial debris facilitate the polarization of infiltrating macrophages toward the M1 phenotype (59). M1 macrophages associated with the early inflammatory phase are highly phagocytic and participate in the inflammatory phase by producing large quantities of pro-inflammatory cytokines (i.e., TNF-α), proteases, and ROS with the ultimate goal of pathogen control and removal of necrotic cell and tissue debris (57, 59).

The late inflammatory phase, which occurs at 5–7 days post-injury, is marked by an accumulation of apoptotic as opposed to necrotic cells, which upon phagocytosis facilitate the polarization of macrophages toward the immunomodulatory M2 phenotype (59). During the granulation tissue formation and wound contraction phase of cutaneous wound healing at 7–10 days post-injury, paracrine effector molecules, including cytokines such as IL-10 and growth factors such as VEGF, PDGF-β, and TGF-β, produced by M2 macrophages recruit fibroblasts into the wound site and promote myofibroblast differentiation (6, 60). M2 macrophages continue to release anti-inflammatory and pro-angiogenic factors, which facilitate the resolution of inflammation, recruitment of endothelial cells, and deposition of new ECM (61, 62). Activated myofibroblasts bridge the wound gap and develop contractile forces to facilitate wound contraction. Growth factors produced by M2 macrophages and myofibroblasts synergistically promote the proliferation and migration of keratinocytes to facilitate wound re-epithelialization (60, 61).

The matrix deposition and tissue remodeling phase, which occurs after 10 days post-injury, is marked by a decrease in macrophage numbers populating the wound site, along with an overall decrease in total cellularity. Granulation tissue formation reaches a plateau and tissue present within the wound site is partially remodeled into fibrotic scar tissue at this time (56, 57).

The above stages of cutaneous wound healing are carefully regulated by the activity of responding macrophages. Similar to the M1/M2 macrophage paradigm associated with the skeletal muscle injury response, cutaneous wound healing is dependent upon a heterogeneous macrophage population and an M1 to M2 phenotypic transition. The pro-inflammatory activity of M1 macrophages during the early inflammatory phase is required for efficient pathogen control. Conversely, M2 macrophage activity during the late inflammatory phase is required for the resolution of inflammation and the recruitment of cells, which facilitate granulation tissue formation and wound re-epithelialization. Perturbation of the M1 macrophage phenotype during the early inflammatory phase, either by conditional depletion or due to impaired recruitment, results in delayed granulation tissue formation and wound closure (63, 64). Similarly, prolonging the M1 macrophage phenotype (i.e., preventing the phenotypic transition to M2) through the exogenous addition of TNF-α during the late inflammatory phase also resulted in poor wound remodeling outcomes (65). Depletion of M2 macrophages during the late inflammatory phase results in prolonged inflammation and impaired wound repair (63, 66). These M2 macrophage depleted cutaneous wounds resemble chronic wounds typically associated with the pathogenesis of chronic venous ulcers (CVU) and diabetes. In fact, studies have shown that failure of cutaneous wound macrophages to undergo the M1 to M2 phenotypic transition represents a hallmark of these chronic inflammatory diseases (65, 67, 68). Taken together, these studies show the importance of functional macrophage heterogeneity and the extent to which immunomodulatory effects of M2 macrophages are critical for efficient wound healing and tissue remodeling.

### The M1/M2 paradigm during the CNS injury response

The role of M1 and M2 macrophages following injury in the central nervous system (CNS) is more ambiguous when compared to other tissues, and is made more complex by the presence of the blood–brain barrier. However, similarities to the macrophage heterogeneity associated with the injury response in other tissues are increasingly being reported.

Central nervous system resident macrophages, referred to as microglia, have long been considered the primary responders to injury in the CNS with little to no role having been recognized for circulating cells until recently. Generally, microglia are recruited to, and form a dense barrier around, the lesion site immediately following spinal cord injury (69, 70). These activated microglia produce large quantities of cytotoxic factors and pro-inflammatory cytokines including IL-1β, IL-6, and TNF-α. This pro-inflammatory response facilitates pathogen control and debris clearance, and also the recruitment of neutrophils and blood-derived monocytes and macrophages (5, 71–73); however, this response is also commonly cited as a driver of poor remodeling outcomes following injury in the CNS. As with other tissue injury responses, functional remodeling following CNS injury involves a transition from a pro-inflammatory to an immunoregulatory and homeostatic response. It remains unknown if microglia show M1 to M2 phenotypic plasticity similar to that observed to monocyte-derived macrophages. However, recent studies suggest that CNS microglia drive an early pro-inflammatory response, but infiltrating macrophages from the circulation may facilitate the M2-like tissue remodeling response (74). Specifically, recruited blood-derived macrophages, showing an anti-inflammatory phenotype consistent with M2 polarization, do not directly enter the lesion center, but are found around the lesion site at 3 days post-injury (74, 75). These immunoregulatory macrophages have been shown to arrive at the site of injury by specifically trafficking through a remote blood–cerebrospinal-fluid (CSF) barrier, the brain ventricular choroid plexus (CP) (8). Once at the injury site, these M2 macrophages produce IL-10 for mitigation of the pro-inflammatory response and contribute to repair mechanisms including remyelination (8, 75, 76).

The participation of M2 polarized macrophages in the CNS injury response is essential to the repair process. For example, the endogenous partial recovery which can be observed following spinal cord injury is abrogated when M2-like macrophages are depleted using antibodies or conditional ablation (74, 75). Consistent with this notion, blockage of CP mediated macrophage trafficking inhibits M2 macrophage recruitment and subsequently impaired recovery following injury (76). These studies show the importance of a heterogeneous macrophage response to CNS injury and, specifically, that M2 macrophages contribute to processes beyond inflammation.

The above studies support an emerging dogma of effective recovery from tissue injury in which initial responses consisting predominantly of M1 macrophages and secondary or later stages consisting predominantly of M2 macrophages drive functional remodeling outcomes. Furthermore, it is appears that M2 macrophages contribute to more than immunomodulation during the response which follows tissue injury. Several organ systems, in addition to the above examples of muscle, skin, and CNS tissue have now been shown to undergo similar responses following injury and are also characterized by heterogeneous and temporally shifting macrophage phenotypes.

## A Departure from the “Classical Paradigm”

The observation of dichotomies in macrophage phenotype in disease pathogenesis as well as tissue remodeling following injury represents a departure from the classical understanding of the macrophage as a primarily phagocytic and pro-inflammatory cell. The foreign-body reaction (FBR) has been well studied over the last three decades (77). Logically, this response is an extension of normal wound healing as the implantation of a biomaterial necessarily requires the creation of a surgical injury. The seminal works in this area by Anderson (77, 78) and others describe the host response to implanted materials as occurring in stages including injury (implantation), protein adsorption, acute inflammation, chronic inflammation, FBR, granulation tissue formation, and encapsulation. These processes are well recognized to be dominated by mononuclear cells, and macrophages in particular.

During a FBR persistent inflammatory stimuli, such as the presence of a non-degradable biomaterial, lead to chronic inflammation and the formation of multinucleated foreign-body giant cells (79). Multinucleate giant cells are formed by the fusion of persistent pro-inflammatory macrophages, consistent with the M1 phenotype, located at the surface of the biomaterial and further exacerbate the deleterious inflammatory response through a process known as “frustrated phagocytosis” (80, 81). Failure to resolve the inflammatory response results in a FBR, leading to the deposition of disorganized fibrous tissue consistent with scaring and encapsulation of the implant (82, 83). This dense fibrous scar isolates the implant and prevents its integration with the surrounding host tissue.

The interpretation of the FBR as a negative occurrence in this context led to the development of materials with a focus on “inertness” and “biocompatibility” (84–86). This focus upon the host response to biomaterials resulted in an associated emphasis upon the material characteristics which determine the host response and downstream outcomes. However, these same characteristics may not be ideal in the setting of regenerative medicine where the focus is upon the restoration of function through the development of new host tissues rather than through the provision of a simple mechanical substitute. These concepts do not imply that medical devices such as hip implants and surgical mesh are not effective for their intended functions, but rather that the intended use, and therefore, the design characteristics and functional requirements, of these materials are incompatible with the goals of regenerative medicine.

The emergence of regenerative medicine and the need and desire for therapies which restore endogenous tissue function has led to a significant increase in our understanding of the role of stem cells in tissue repair as well as innovation and development of new biomaterials as stand alone therapies and/or delivery systems for cells or biologic factors. These materials are most often degradable in nature, and include engineered biologic cues or inherent bioactivity when derived from natural or tissue based sources. As such, the host response to these materials will be significantly different and more complex than the response to mono-component, synthetic or metallic implants. Further complexity is seen when materials are used in combination with cells or other factors.

It is now recognized that certain materials when used alone or in concert with a cellular component can provide an inductive template for constructive and functional tissue remodeling. That is, the provision of a bioactive material and/or cells leads to the formation of new, site-appropriate tissue. One example of such materials is biologic scaffolds composed of ECM (87, 88). These materials are derived through the decellularization of source tissues and organs and are widely utilized in regenerative medicine approaches to tissue reconstruction (89, 90). By the nature of the source (i.e., intact tissue), the materials that result from efficient decellularization can be thought of as degradable reservoirs of tissue specific structural and functional components. These materials have been shown to be effective templates for constructive remodeling in both pre-clinical and clinical applications, and in several body systems (87, 88). However, it should be noted that reports of the effectiveness of ECM based scaffold materials are variable and highly dependent on the methods of scaffold production.

Despite the distinct differences in long-term outcomes which have been observed with various ECM based scaffold materials, all ECM implants have been shown to elicit a histologically similar cellular response in the first week to month post-implantation (3, 91). This response is characterized by an early infiltrate of neutrophils followed by a dense infiltrate of mononuclear cells. Under the classical paradigm, such a response would commonly be associated with progression to a FBR with negative implications for functional tissue remodeling outcomes. However, the response typically proceeds down one of three distinct pathways: (1) a classic FBR with encapsulation and no signs of constructive remodeling; (2) chronic inflammation and degradation or integration of the material with little to no constructive remodeling; or (3) reduction of the inflammatory infiltrate with subsequent constructive remodeling (3, 91).

Based upon these disparate outcomes, it was hypothesized that, though the early host response to the materials was histologically similar (i.e., characterized by a dense infiltration of mononuclear cells in the site of implantation), differences in the early macrophage phenotype to certain ECM scaffold materials might exist and that these differences may be related to downstream remodeling outcomes. Indeed, this hypothesis was shown to be correct with those ECM scaffolds which elicited constructive remodeling outcomes being associated with a timely transition from an M1 to an M2 macrophage phenotype (3, 92, 93). These studies have provided the impetus for investigation of macrophage phenotype in a number of regenerative medicine applications using biomaterials and cell-based therapies. The results of these investigations now clearly show a correlation between macrophage phenotype and successful outcomes associated with multiple regenerative medicine strategies. A review of selected studies which demonstrate this phenomenon are described below with a focus upon multiple strategies (materials, cells, and bioactive factors) which show associations between macrophage phenotype and remodeling outcomes.

### The M1/M2 paradigm in tissue engineering and regenerative medicine

An endogenous host injury response, consisting of immunomodulation, including the participation of M2 type macrophages represents a necessary component of efficient and functional tissue repair. It is therefore logical that regenerative medicine strategies aimed at activating or augmenting endogenous repair mechanisms should utilize a similar strategy. Regenerative medicine strategies aimed at promoting M2 macrophage activation have included cell-therapy and the implementation of synthetic and biologic scaffold materials, among others.

### Cellular therapy

Cellular therapy is generically defined as the transplantation or delivery of exogenous cells to sites of injured or missing tissues. Stem and/or progenitor cells are often used in regenerative medicine applications because of their multi-lineage differentiation potential and well-recognized resistance to oxidative stress (94, 95).

The cell source is most commonly autologous due to immune rejection considerations, although many studies are investigating the use of allogeneic sources. Cellular therapy based strategies aimed at promoting tissue remodeling have been used to treat injured tissues including the myocardium, the spinal cord, and skeletal muscle, among others. Despite moderate pre-clinical and clinical success, cell-therapy is associated with limitations including failure of the exogenous cells to engraft within host tissue (96–101). It is now increasingly recognized that therapeutic outcomes associated with cellular therapy are largely a result of paracrine effects exerted by the transplanted or delivered cells upon the injured host tissue microenvironment rather than direct differentiation of the transplanted cells into new tissues (102–107). These paracrine effects include modulation of macrophage polarization and beneficial remodeling events facilitated by a transition to the M2 macrophage phenotype (108–110).

Co-culture experiments have shown that the secreted products of different stem/progenitor cells directly promote an M2 macrophage phenotype (111–113). A large number of pre-clinical studies also support these results. For example, following spinal cord injury, transplanted bone marrow-derived mesenchymal stem cells (MSC) modulate the host inflammatory microenvironment by promoting an M1 to M2 transition, which ultimately leads to a permissive environment for axonal extension and functional recovery (114). Furthermore, following traumatic brain injury, intravenous (IV) delivery of multipotent progenitor cells promotes the polarization of microglia to an M2-like phenotype (115). Several studies, using cells of multiple origins (i.e., autologous and allogeneic bone marrow-derived MSCs, adipose derived MSCs, and umbilical derived MSCs, among others), suggest that the therapeutic effects associated with exogenous cell delivery for the treatment of myocardial infarction are a result of enhanced macrophage polarization switching (116–118). Cellular therapy mediated M2 macrophage polarization has been used to promote tissue remodeling and repair in several anatomic locations and disease states including kidney ischemia-reperfusion injury and asthma associated alveolar inflammation, among others (119–121).

### Scaffold materials

Regenerative medicine strategies aimed at promoting tissue reconstruction or replacement often employ the use of surgically implantable synthetic or biologic materials designed to serve as cellular support scaffolds. As described above, implantation of these materials following injury alters the default injury response. For example, following surgical placement, synthetic and/or biologic scaffold materials are able to affect the phenotype of infiltrating inflammatory cells, host progenitor cell activity, as well as fibrosis and fibrous capsule development (78, 122). These effects depend on the scaffold composition, degradability, cellularity, porosity, and implantation site among others (78).

### Synthetic scaffold materials

As stated above, the surgical placement of non-degradable synthetic scaffold materials is commonly associated with a FBR consisting of persistent M1 macrophage activity and an increased deposition of scar tissue (3, 78, 91, 123–125). Recently, strategies aimed at modulating material properties to reduce the persistent pro-inflammatory M1 macrophage response to synthetic biomaterials have been examined. These strategies have included alterations in scaffold surface chemistry and structural characteristics. However, some of the studies examining these strategies are associated with conflicting results. For example, one study suggests that synthetic scaffold materials composed of fibers with smaller diameters are associated with more M2-like macrophage activation when compared to their larger diameter counterparts (126). In contrast, another study showed that larger fiber diameter enhanced M2 macrophage polarization (127).

A recent series of studies has demonstrated the effects of material pore size upon integration of the material as well as macrophage phenotype (13, 128–131). In these studies, materials were produced with tight distributions of pore sizes. Results showed that materials possessing pores of roughly 30–40 μm were shown to integrate with reduced encapsulation and higher vascularity when implanted into dermis or cardiac tissues (13, 131). These changes in outcome were also associated with shifts in macrophage phenotype (128, 131). However, interestingly, the shifts in phenotype were observed to be spatially distinct with cells outside of the pore templated implants having an increased M2 phenotype as compared to non-porous implants, and the cells within the implant having a predominantly M1 phenotype (128). These studies suggest that manipulation of the structural and surface characteristics of synthetic scaffold materials can affect macrophage phenotype. Specifically, some of these manipulations appear to alter the macrophage response and are also associated with improved outcomes.

Another manner in which biomaterials can be tailored to promote shifts in macrophage phenotype is through the use of biologically active molecules such as growth factors and cytokines. Examples of these approaches are numerous and are commonly employed in regenerative medicine with resulting improvements in remodeling outcomes. A recent study investigated the effects of incorporation of either M1 (IFN-γ) or an M2 (IL-4) promoting cytokines within a polysulfone tube upon nerve growth across a gap defect when the tubes were used as guidance conduits (132). The results of the study demonstrated that polarization of macrophages toward a more M2 phenotype was associated with increased Schwann cell infiltration and neurite outgrowth. These effects were further examined *in vitro*, with results suggesting that macrophage derived factors were at least in part the cause of the observed chemotaxis of Schwann cells.

### Biologic scaffold materials

The biologically derived scaffold materials used in regenerative medicine applications are sourced from a variety of natural sources including mammalian tissues as well as plant, insect, and bacterial sources. These materials offer the inherent advantage of the native ligand landscape and bioactivity resulting from their source material. This inherent bioactivity also leads to added complexity in the host response to these materials. Among these materials, scaffolds derived from mammalian tissues represent the most commonly used materials in pre-clinical and clinical regenerative medicine applications (133–135).

Biologic scaffold materials composed of mammalian ECM have been used to promote constructive tissue remodeling in a variety of clinical applications including hernia repair, rotator cuff reconstruction, esophageal preservation, and skeletal muscle replacement, among others (88, 136, 137). ECM bioscaffolds are derived through the decellularization of mammalian tissue (89, 90, 138). The most common tissue sources are xeno- or allogeneic in nature and include decellularized dermis, small intestine, bladder, and pericardium among others.

It is now well recognized that the ability of these materials to promote constructive remodeling is tied to their ability to modulate the host macrophage response (3, 92, 93, 135). Multiple studies have shown that ECM based scaffold materials which are properly prepared facilitate a transition from an M1 to an M2 phenotype around 7–14 days post-implantation (3, 92, 93). The exact mechanisms by which these materials facilitate this response remain largely unknown; however, a number of key aspects have been identified. The materials must be adequately decellularized to remove potentially immunogenic cellular constituents and the material must be able to degrade (3, 92, 93, 139). In the presence of excess cellular material or if the material has been chemically crosslinked to prevent degradation, an extended M1 type immune response with no transition to an M2 response is observed and is associated with poor remodeling outcomes or encapsulation.

The necessity of degradation for the transition to an M2 phenotype suggests that breakdown products of the ECM scaffold material may possess immunomodulatory activity. Studies have shown that ECM bioscaffolds can be solubilized and the degradation products formed into a hydrogel under physiologic conditions (140, 141). This hydrogel ECM, when used as a coating for polypropylene surgical mesh, can facilitate a transition from the default M1 and FBR type response to a more M2 type response with a reduction in the FBR and encapsulation (142). These results, as well as other recent *in vitro* studies, further demonstrate the inherent immunomodulatory nature of ECM based biomaterials as well as their ability to improve remodeling outcomes (124, 142).

## Words of Caution

The above examples clearly illustrate an emerging paradigm in regenerative medicine. That is, strategies which are able to modulate the host response from an M1 to an M2 macrophage response are associated with better outcomes. However, these results should be interpreted with caution. Macrophage phenotypes have been described in many ways (143). “M1” and “M2” (with M2 macrophages including subsets M2a, M2b, and M2c) represents the common terminology used to describe these cells. Macrophage phenotypes have also been described as a spectrum between M1 and M2 with any individual cell being capable of expressing multiple aspects of either phenotype at any given time. Given this phenotypic heterogeneity, and the transient nature of the remodeling process following injury, further study of biomaterials-macrophage and stem cell-macrophage interactions are warranted, as is more thorough definition of the resultant phenotypes and their unique functions. It is unlikely that macrophages which result from interactions with biomaterials, particularly those with inherent naturally occurring ligand landscapes, or stem cells will possess phenotypes which precisely resemble the canonical IFN-γ and LPS (M1) or IL-4, IL-13 (M2a), IC and TLR/IL1-R ligand (M2b), or IL-10 (M2c) activated macrophages.

Adding further complexity to the definition of macrophage phenotypes in regenerative medicine applications is the variability in tissue resident macrophage populations. For example, microglia are the resident macrophages of the brain and derive from the embryonic yolk sac during development and persist in the brain thereafter, presumably through a process of local replication (144–146). As is described above, these cells have been demonstrated in a number of studies to have phenotypes which are distinct from circulating monocyte-derived macrophages and are known to play distinct roles in a number of CNS disease processes. Other tissue resident macrophage populations also exist, each with a distinct and tissue specific phenotype (147). While a full description of tissue resident macrophages in all body systems and their distinct phenotypic characteristics is beyond the scope of this review, it is important to understand how these differences and the relative contributions of local versus circulating cells will affect outcomes.

At present, studies investigating macrophage phenotype following exposure to biomaterial implants have largely relied upon single surface or gene expression markers as indicators of M1 and M2 polarization. It is now well established that macrophages possess highly complex and plastic phenotypes and that the use of multiple phenotypic markers is essential. Further, a better understanding of the functional implications of these phenotypes is needed to create a mechanistic understanding of the ways in which macrophages may direct tissue remodeling outcomes following biomaterial implantation or stem cell delivery. With this understanding, next generation therapies can be developed to target and modulate specific macrophage phenotypes with desirable characteristics for the given application.

It should further be recognized that baseline macrophage polarization states may be affected by patient characteristics. As is mentioned above, there is now increasing evidence for changes in macrophage phenotype and response to activating stimuli with age and disease both acute and chronic (12, 23, 148, 149). Commonly employed pharmacologic interventions may also affect the response. Also, it is logical that the tissue microenvironment which results following an injury may also be different than that which is experimentally and sterilely created in an animal model. Thus, there is a need to investigate macrophage response to regenerative medicine strategies in animal models which, at least in part, can mimic aspects of these complex situations.

## Conclusion

Macrophage polarization has been clearly shown to be an important determinant of success in regenerative medicine strategies for tissue reconstruction. Macrophages can promote both positive and negative outcomes, which are dependent upon the context in which they are encountered, their phenotype, and function. However, at present, there remains much to be investigated and defined regarding macrophage phenotypes associated with biomaterials, stem cells, and regenerative medicine. Thus, context specific definitions and identification of beneficial phenotypes are needed. Similarly, the unique functions of these cells must also be clearly defined in order to better understand their true role in the remodeling process. Indeed, a focus upon macrophage function during the process of constructive remodeling may prove more useful than further characterization of complex phenotypic markers. Moving studies from correlative to causative and expanding the number of outcome metrics, both phenotypic and functional, will assist in defining both biomaterials and stem cell associated phenotypes and also provides targets for next generation regenerative medicine therapies, which seek to modulate macrophages as a means of promoting functional tissue recovery – a macrophage centric approach. It is increasingly clear that those strategies that adopt such an approach to regenerative medicine will meet with improved success.

## Conflict of Interest Statement

The authors declare that the research was conducted in the absence of any commercial or financial relationships that could be construed as a potential conflict of interest.
